# A narrative review of factors influencing detection and treatment of depression in Vietnam

**DOI:** 10.1186/1752-4458-7-15

**Published:** 2013-05-06

**Authors:** Maria Niemi, Mats Målqvist, Kim Bao Giang, Peter Allebeck, Torkel Falkenberg

**Affiliations:** 1 Karolinska Institutet, Department of Public Health Sciences, Tomtebodavägen 18A, 17165, Stockholm, Sweden; 2 Department of Women’s and Children’s Health, Uppsala University, Drottningg. 4, 751 85, UPPSALA, Sweden; 3 Hanoi Medical University, Hanoi, Vietnam; 4 Karolinska Institutet, Department of Public Health Sciences, Tomtebodavägen 18A, 17165, Stockholm, Sweden; 5 Karolinska Institutet, Department of Neurobiology, Care Sciences and Society, Division of Nursing, Unit for Studies of Integrative Care, Stockholm, Sweden & IC – The Integrative Care Science Center, Järna, Sweden

**Keywords:** Vietnam, Depression, Mental health system, Depression screening, Depression treatment

## Abstract

Depression is among the most common psychiatric conditions in primary health care, and constitutes an important part of the global disease burden. However, it is difficult to obtain comparable data on depression worldwide and models for treatment and intervention need to be locally adapted. We conducted a narrative review of research literature on factors that influence depression screening, diagnosis and treatment among the Vietnamese population. This explorative approach included studies describing: a) culturally or contextually specific risk-factors for depression; b) any depression treatment seeking or treatment acceptability/adherence aspects or; c) depression screening among Vietnamese patients. We searched the PubMed and Cinahl databases, as well as relevant Vietnamese peer-reviewed journals and this produced 20 articles that were included in the review. Our findings indicate the importance of considering somatic symptoms when screening for depression in Vietnam as well as the use of culturally adapted and dimensional screening instruments. Our study confirms that depression reflects chronic social adversity, and thus an approach to mental health management that focuses solely on individual pathology will fail to address its important social causes. Further studies should elucidate whether neurasthenia is a commonly used illness label among Vietnamese patients that coincides with depression. The tendency among Vietnamese to seek traditional Vietnamese medicine and meditation practice when experiencing emotional distress was supported by our findings.

## Introduction

Depression is among the most common psychiatric conditions in primary health care, and constitutes an important part of the global disease burden. Depressive disorder is increasingly recognized as a major global problem and was estimated to account for 2,5% of total DALY’s in the year 2010 [1]. Increased attention has been paid to this, and Crisholm et al. [2] used data from 14 regions in the world to analyse cost-effective ways of reducing the burden. They found that it is difficult to obtain comparable data on depression worldwide and that models for treatment and intervention need to be locally adapted.

In this paper we will review existing literature to study what needs to be taken into consideration regarding depression diagnosis and treatment in Vietnam. The study is part of a project aiming to implement locally appropriate mental health intervention models at the community level.

### Cultural background and mental health system in Vietnam

In Vietnam, Confucianism, Buddhism and Taoism have carried major impacts in creating a holistic thinking where clear distinctions between physical and psychological symptoms are not made [3]. The Cartesian mind/body dualistic framework that underlies Western psychiatric nosology does thus not necessarily concur with this holistic view [3]. The persistence of a traditional model of health, illness and the body among lay people has been described as the perhaps most salient fact in Vietnamese medical history [4]. The Vietnamese are found to often seek care from traditional approaches, including Traditional Vietnamese Medicine (TVM), Traditional Chinese Medicine, witchcraft, spiritual blessing and sorcery [3].

There are 64 provinces in Vietnam and in 27 of these there is a separate psychiatric hospital, while in the rest of the provinces psychiatric problems are handled at the district hospital. There are three mental hospitals that operate under the Ministry of Health (MoH) [5]. Two provinces do not have a mental hospital or department [6]. The medical management of mental illness in Vietnam only involves medication, and there is no family education or psychotherapy. Those psychologists who work in hospitals are mainly engaged in clinical testing [5]. Twenty percent of the physician-based primary health care services include complementary/alternative/traditional practitioners. TVM is used for neurasthenia, and dissociative disorders and treatment consists mainly of acupuncture, massage and herbal medicines. Patients with schizophrenia, personality disorders, paranoia, or suicidal thoughts appear not to be treated by TVM [5].

In the past, medical care in Vietnam was free at all levels. However, after the adoption of the economic renovation policy in 1986, only a part of patients’ medical costs have been covered by public funding, while private clinics have opened. Mental hospitals are entirely subsidized by the government [5]. The government only pays for control and medication of epilepsy and schizophrenia, while medication and treatment for other mental illnesses is paid out-of-pocket. The supply and pricing of psychiatric medicines is regulated by the government, and the cost of one day’s antipsychotic medication is 33% of one day’s minimum wage [6].

Vietnam’s mental health plan was last revised in 2010. The eaqrlier version consisted of a national plan of action on treatment of schizophrenia and epilepsy in hospitals [7] but the revised plan also comprises the integration of mental health services into primary care [8], as well as the treatment of depression [9]. The human resources for mental health are scarce in Vietnam. In 2011, there were 1.01 psychiatrists per 100 000 population, 67,39 medical doctors not specialised in psychiatry, 75.34 nurses and 0.03 psychologists working in the mental health sector [8]. Researchers and policy makers have jointly identified the main gaps in the Vietnam mental health system to be the lack of knowledge about the feasibility and cost of any intervention [7].

## Methods

We utilized a modified version of the procedure described by Green and co-workers [10] to conduct a narrative review of factors that influence depression screening, diagnosis and treatment among the Vietnamese population. The aim was to find published research that describes factors of importance for developing appropriate depression screening and treatment methods. This explorative approach included studies describing: a) culturally or contextually specific risk-factors for depression (of relevance for treatment strategies); b) any depression treatment seeking or treatment acceptability/adherence aspects (of relevance for treatment or screening strategies) or; c) depression screening among Vietnamese patients (of relevance for screening strategies). The inclusion criteria were thus purposefully relatively loosely defined. The search terms used included depression, Vietnam*, and excluded veteran (due to the large number of studies found on Vietnam veterans in the USA), in title or abstract, in PubMed and CINAHL (See Table 1). The searches were conducted on December 14^th^, 2012. We also separately searched six relevant Vietnamese peer-reviewed research journals to see if any work had been published in Vietnamese. These searches were conducted between April 17^th^ and 19^th^. We only included studies among specific groups, such as elders, perinatal women/men, or youth when the results were deemed generalizable beyond that group. Also, we included studies with refugees/immigrants in other countries, when the studies were in particular about the cultural expression of depression, or conducted with newly arrived immigrants/refugees. However, studies with Vietnamese patients in other countries that studied health system factors were not included, as they were not deemed relevant for the Vietnamese health care system.

**Table 1:** Search strategy and number of included studies

**Database**	**Date**	**Search terms**	**Hits**	**Included studies**
Medline	2012-12-19	Depression (in title/abstract) AND Vietnam* (in title/abstract), NOT veteran* (in title/abstract)	148	18 studies included
Cinahl	2012-12-19	Depression (in abstract) AND Vietnam* (in abstract), NOT veterans	39 (7 new)	No new studies included
Vietnamese journals: J of Medical Research; J of Medical Practice; J of Clinical Medicine; J of Preventive Medicine; J of Medicine Ho Chi Minh; J of Public Health	2013-04-17 – 2013-04-19	Depression	2 (2 new)	2 studies included

## Results

Two searches were conducted in the two databases, as well as six separate searches in the six Vietnamese journals, and this yielded 157 hits. All the hits were screened through reading the title, abstract or full text article in order to decide whether they fit the search criteria. This screening process left 20 studies. The main study characteristics including study title, authors, setting, sample and methodology are shown in Table 2. The results relevant for the study aim are presented below, reference numbers are annotated in the text as listed in Table 2.

**Table 2:** Included studies for literature review

	**Authors**	**Reference**	**Aim**	**Population and setting**	**Methods**
1.	Nguyen N-L D, Hunt D D, Scott C S	Screening for depression in a primary care setting in Vietnam. (2005) The Journal of Nervous and Mental Disease, vol. 193, n. 2	To assess depression in patients who were already diagnosed with depression by a Vietnamese psychiatrist and in patients presenting at a primary care clinic.	115 clinically depressed patients at the Center for Mental health and 177 patients at a primary care clinic in Ho Chi Minh City.	Depression assessed using the Vietnamese Depression Scale.
2.	Steel z, Silvoe D, Giao N M, Phan T T B, Chey T, Whelan A, Bauman A, Bryant R A	International and indigenous diagnoses of mental disorder among Vietnamese living in Vietnam and Australia. (2009) The British Journal of Psychiatry, vol. 194, pp: 326-333	To investigate the prevalence of Western and indigenously defined mental disorders among Vietnamese living in Vietnam and Australia, comparing data with an Australian-born sample.	Population survey among 3039 Vietnamese living in Mekong Delta region, 1161 Vietnamese living in Australia and 7961 Australian-born, living in Australia.	Western defined mental disorders diagnosed with the Composite International Diagnostic Interview and the Vietnamese indigenously derived Phan Vietnamese Psychiatric Scale. Assessment of functional impairment and service use.
3.	Phan T, Steel Z, Silvoe D	An ethnographically derived measure of anxiety, depression and somatization: The Phan Vietnamese Psychiatric Scale. (2004) Transcultural Psychiatry, vol. 41, pp: 200-232	To develop and validate the Phan Vietnamese Psychiatric Scale	Vietnamese literature on traditional medicine, folk-medicines, novels, folktales, fairytales, poetry, reviews of traditions, cultures, customs and religions. 180 Vietnamese refugees and immigrants living in Sydney	Identification of idioms of distress through Vietnamese literature and ethnography for scale development, Scale validation through estimates of Internal Consistency, confirmatory factor analysis and multitrait-multimeasure analysis.
4.	Fry A J, Nguyen T	Culture and the self: implications for the perception of depression by Australian and Vietnamese nursing students. (1996) Journal of Advanced Nursing, vol. 23, pp:1147-1154	To test certain theoretical assumptions of self and depression by measuring perceptions of depression.	94 australian nursing students from Australia, and 93 Vietnamese nursing students from Ho Chi Minh City.	Survey with a vignette case of depression in a family context and a list of depression symptoms without a context. Participants were asked to rate the two depression cases with the Hopkins Symptom Checklist – 25
5.	Groleau D and Kirmayer L J	Sociosomatic theory in Vietnamese immigrants’ narratives of distress. (2004) Anthropology & Medicine, vol. 11, pp:117-133	To explore reasons for the under-utilization of mental health services by Vietnamese immigrants to Canada.	18 Vietnamese immigrants in Canada, identified through a community survey because they reported four or more somatic, emotional, or medically unexplained symptoms and had not used any mental health service for their problem.	Ethnographic interviews using the McGill Illness Narrative Interview.
6.	Hinton D H, Pham T, Tran M, Safren A S, Otto M W, Pollack M H	CBT for Vietnamese refugees with treatment-resistant PTSD and panic attacks: A pilot study. (2004) Journal of Trauma and Stress, vol. 17, pp: 429-433	To examine the acceptability, acceptability and therapeutic efficacy of a culturally adapted CBT for Vietnamese refugees	12 Vietnamese refugees with treatment-resistant PTSD and panic attacks.	Treated in two separate cohorts of six, with staggered treatment onset, outcomes measured with Harvard Trauma Questionnaire, Anxiety sensitivity Index, Hopkins Symptom Checklist-25 anxiety and depression subscales.
7.	Dinh T Q, Yamada A M, Yee B W K	A culturally relevant conceptualization of depression: an empirical examination of the factorial structure of the Vietnamese Depression Scale. (2009) International Journal of Social Psychiatry, vol. 55: 496	To empirically derive the factorial structure of the Vietnamese Depression Scale to support its use as a culturally responsive depression screening tool in community samples of Vietnamese adults.	Community sample of 180 Vietnamese refugee adults in the USA	Vietnamese Depression Scale interviews conducted to examine its factorial structure, reliability and associations with recognized socio-demographic correlates.
8.	Cheung F and Lin K-M	Neurasthenia, depression and somatoform disorder in a Chinese-Vietnamese woman immigrant. (1997) Culture, Medicine and Psychiatry, vol. 21, pp:247-258	To provide an in-depth ethnographic case study of a Chinese-Vietnamese immigrants illness history and cultural formulation.	28-year-old Chinese-Vietnamese female, immigrant in the USA, enrolled through a neurasthenia and chronic fatigue syndrome study	Ethnographic case study interview.
9.	Niemi M, Falkenberg T, Nguyen M T, Patel V, Faxelid E	The social contexts of depression during motherhood: a study of Explanatory Models in Vietnam. (2010) Journal of Affective Disorders, vol. 124, pp: 29-37	To elicit illness explanatory models of depression and postnatal depression from mothers and health workers who meet mothers during their pregnancy and/or postpartum period.	Nine mothers and nine health workers from a semi-rural community in north Vietnam.	Illness explanatory model interviews using a case vignette of depression and postnatal depression.
10.	Tran TD, Tran T, La B, Lee D, Rosenthal D, Fisher J.	Screening for perinatal common mental disorders in women in the north of Vietnam: a comparison of three psychometric instruments (2011) Journal of Affective Disorders vol. 133, pp: 281-293	To establish the validity of three widely used psychometric screening instruments in detecting CMDs in women in northern Viet Nam.	A community-based representative cohort of 364 Vietnamese women in the perinatal period, in the north of Vietnam.	Translated and culturally verified versions of the Edinburgh Postnatal Depression Scale, General Health Questionnaire 12 items, Zung's Self-rated Anxiety Scale and the Structured Clinical Interview for DSM IV were administered. Calculation of area under ROC Curve and Cronbach’s alpha.
11.	Buchwald D, Manson S M, Breneman D L, Dinges N G, Keane E M, Beals J, Kinzie J D	Screening for depression among newly arrived Vietnamese refugees in primary care setting (1995) Western Journal of Medicine, vol. 163, pp: 341-345	To describe the use of the Vietnamese Depression Scale to examine the nature and extent of depression among adult Vietnamese refugees seen in a primary health care clinics within two months of their immigrating to the United States.	1998 ethnic Vietnamese refugees 16 years of age or older, recruited from ten refugee health clinics	The Vietnamese Depression Scale was incorporated into the clinic intake procedure, which also elicited information about socioeconomic background, medical history and reason(s) for the visit.
12.	Hinton W L, Du N, Chen Y-C J, Tran C G, Newman T B, Lu F G	Screening for major depression in Vietnamese refugees: A valisdation and comparison of two instruments in a health screening population (1994) Journal of General Internal Medicine, vol. 9, pp: 202-206	1) Using standard cut-offs, to determine the accuracy of two instruments for major depression in a nonpsychiatric clinic population of Vietnamese refugees; 2) to examine the utility of other cut-offs; 3) to compare the overall accuracies of the two instruments using Receiver Operating Characteristic (ROC) curve analysis	206 newly arrived (6 months or less) Vietnamese refugees between the ages 18 and 65 years undergoing routine, mandatory, health screening at the San Francisco General Hospital Refugee Medical Clinic	All participants completed the Structured Clinical Interview for DSM-III-R, administered by a psychiatrist, and completed the Indochinese Hopkins Symptom Checklist Depression Subscale, the Anxiety Disorder Interview Schedule for Posttraumatic Stress Disorder and the Vietnamese Depression Scale.
13.	Kinzie J D, Manson S M, Vinh D T, LAn N T T, Anh B, Pho T N	Development and validation of a Vietnamese-language depression rating scale (1982) Americal Journal of Psychiatry, vol. 139, pp: 1276-1281	1)Describe the development and specific items of a Vietnamese-language depression scale derived from the Vietnamese culture; and 2) report the results of the validation of this scale; and 3) discuss the implications of this scale for further clinical research and the cross-cultural study of depression.	Development of scale by research team including a psychiatrist who had lived in Vietnam, an anthropologist and four Vietnamese mental health workers. Pretest done with 20 Vietnamese adults. Validation done by comparing result in two groups; a psychiatric clinic index group meeting DSM-III-R criteria for major depression from the Oregon Health Sciences University Indochinese Psychiatric Clinic (N = 21) and a matched community sample (N=44).	The Vietnamese Depression Scale was developed by the first group, pretested with the second group for clarity and consistency, and then tested with the third group.
14.	Tran T D, Tran T, Fisher J	Validation of three psychometric instruments for screening for perinatal common mental disorders in men in the north of Vietnam (2012) Journal of Affective Disorders vol. 136, pp: 104-109	To validate the Edinburgh Postnatal Depression Scale (EPDS), the Zung Self-rated Anxiety Scale (Zung SAS) and the General Health Questionnaire 12 Items (GHQ 12) for use with men whose partners were pregnant or had recently given birth	231 eligible men whose partner was at least 28 weeks pregnant or were mothers of 4-6 week old babies and registered at selected commune health stations in Ha Nam and Hanoi	EPDS, Zung-SAS and GHQ-12 scores collected through structured clinical interview were compared against the Structured clinical interview for DSM-IV axis 1 diagnoses of depression, generalized anxiety and panic disorder, administered by a psychiatrist.
15.	Guindon G E, Boyle M H	Using anchoring vignettes to assess the comparability of self-rated feelings of sadness, lowness of depression in France and Vietnam (2012) International Journal of Methods in Psychiatric Research, vol. 21, pp: 29-40	To estimate the extent to which mental disorders such as depression are influenced by health expectations could have important implications for our understanding of these conditions as well as their role in accounting for disability	Probability sampling used to select one respondent per household in France (3490) and Vietnam (N=1001). Substudy with vignettes done with subgroup of French (N=251) and Vietnamese (N=856) participants	Frequency of depressive disorders examined by sex with the International Classification of Diseases, 10^th^ revision (ICD-10). Vignette based questions about mental health were posed to the subgroups, where they were asked to rate their own health status as extreme, severe, moderate, mild and none in relation to the vignettes. The results of the two assessment procedures were compared.
16.	Fisher J R W, Tran H T T, Tran T	Relative socioeconomic advantage and mood during advanced pregnancy in women in Vitenam (2007) International Journal of Mental Health Systems, vol. 1:3	To investigate the prevalence and determinants of depression in a cohort of pregnant women	61 women in their second half of pregnancy recruited from the National Obstetric Hospital in Hanoi. Only women who appeared confident, more advantaged and well-educated and therefore more willing to discuss sexuality were approached, as the study subsidiary aim was to discuss sexual beliefs and behaviours.	The self-report questionnaires Edinburgh Postnatal Depression Scale (EPDS) and the Intimate Bonds Measure (IBM) were completed by all participants.
17.	Fisher J R W, Morrow M M, Ngoc N T N and Anh L T H	Prevalence, nature, severity and correlates of postpartum depressive symptoms in Vietnam (2004) International Journal of Obstetrics and Gynaecology, vol. 111, pp: 1353-1360	To examine depressive symptomatology in women after childbirth in Ho Chi Minh City	506 women were recruited consecutively in the postnatal wards of Hung Vuong Obstetrics and Gynaecology Hospital and maternal and Child Health and Family Planning Center, and invited to take part in the study at the first clinic visit.	Individual structured interviews about health and social circumstances, including the EPDS were administered during clinic visits
18.	Fisher J, Tran T, Tran T D, Dwyer T, Nguyen T, Casey G J, Simpson J A, Hanieh S, Biggs B-A	Prevalence and risk factors for symptoms of common mental disorders in early and late pregnancy in Vietnamese women: A prospective population-based study (2012) Journal of Affective Disorders, e-pub ahead of print	To establish the prevalence and psychosocial risk-factors for clinically significant symptoms of CMD in early and late pregnancy in women in rural Vietnam.	A population-based sample of 497 women, recruited from all pregnant women in randomly selected communes in Ha Nam province, were surveyed in early and late pregnancy.	Common mental disorders were assessed using the Edinburgh Depression Scale (EDS). Coincidental life adversity was assessed with a single question, and the quality of intimate partner relationship was assessed with the Intimate-Bond Measure –Vietnam (IBM-V)
19.	Huong, N T, Anh L V, Dunne M	Validity and reliability of two scales measuring depression and anxiety for community research use among youth in the community (2010) Journal of Public Health (Vietnamese), vol. **7**, pp: 25-31	To validate the self-reported CES-D (The Center for Epidemiological Studies-Depression Scale) among Vietnamese adolescents and; to develop and conduct a preliminary validation of a self-reported short form anxiety scale for adolescents.	299 school students aged 13-18 years in Chi Linh and Dong Da districts in Hanoi	Both scales had good internal consistency (anxiety scale: α = 0.82 and CES-D: α = 0.87), and have good psychometric properties among the Vietnaese adolescent population studied.
20.	Thanh N N, Linh L C	Validity and reliability of the depression scale for the youth and adolescent and related factors in Chi Linh district, Hai Duong province (2010) Journal of Public Health (Vietnamese), vol. 16, pp: 33-41	To develop and measure the validity and internal consistency reliability of the Centre for Epidemiological Studies - Depression Scale (CES-D) in seven towns/communes of Chi Linh district, Hai Duong province, and; to identify the relationship between sociodemographic variables and the mean score obtained on the depression scale.	The CES-D was translated and adapted for the Chi Linh district context. The revised scale was then tested among 12,447 youths and adolescents (age 10-24 years)	The scales internal consistency (cronbach's alpha) was 0.82. All tested socio demographic variables (gender, marital status, age, education level and urban or rural residence) were significantly associated with the total mean score of depression.

### Screening

We found studies reporting the development, assessment and use of two Vietnamese psychiatric scales, the Vietnamese Depression Scale (VDS) and the Phan Vietnamese Psychiatric Scale. The VDS was developed in the USA to be used among newly arrived refugees, and includes some psychophysiological symptoms derived from DSM-III-R, as well as some specific Vietnamese descriptions of cognitive, affective, and somatic indications of depression (12). The scale contains 15 items with a maximum score of 34 (12). When comparing the Indochinese Hopkins Symptom Checklist Depression Subscale (HSCL-D) with the VDS, they were found to have similar areas under ROC curve (0.91 and 0.93 respectively), when measured at the optimal cutoffs of 26 and 11 respectively. These cut-offs gave Se 96%, Sp 86% and ppv 60% for the HSCL-D and Se 98%, Sp 79% and ppv 79% for the VDS (12).

In the development of the Phan Vietnamese Psychiatric Scale (PVPS), some ethnographic and qualitative research was undertaken. Through interviews with 180 Vietnamese community residents in Sydney and review of Vietnamese literature, the scale was derived from Vietnamese idioms, expressions and understandings of mental illness. Many of the symptoms were derived from Vietnamese health beliefs regarding energy flow and the location of emotional states in physical parts of the body (3). When comparing two diagnostic instruments for mental disorder among Mekong Delta Vietnamese and Vietnamese living in Australia, it was found that the PVPS detected four times more cases of mental illness among Mekong Delta Vietnamese than the internationally used CIDI (2). A validation of three screening instruments, the EPDS, the Zung-SAS and the GHQ-12 for the detection of common mental disorders (depression, anxiety and panic) among perinatal women and men in northern Vietnam discovered that the valid cut-off points in that setting are much lower than in most other countries (10). The validated cut-off scores for women were 3/4 (Se 69.7%; Sp 72.9%), 37/38 (Se 67.9%; Sp 75.3%) and 0/1 (Se 77.1%; Sp 56.6%) (10) and for men were 4/5 (Se 68.3%, Sp 77.4%, ppv 75.8%), 35/36 (Se 70.7%, Sp 79%, ppv 77.5%) and 0/1 (75.6%, Sp 74.7%, ppv 74.9%) (14). The authors discussed that this is probably due to differences in emotional literacy, non-familiarity with test-taking and the effects of chronic social adversity (10, 14). Two Vietnamese language studies presented the validation (19, 20) and internal consistency (20) of the Center for Epidemiological Studies-Depression Scale (CES-D) among adolescents and young adults. The scale was validated with good results, and the internal consistency (cronbach's alpha) was found to be 0.82 (20) and 0.87 respectively (19).

### Symptomatic presentation and risk-factors

Seven of the retrieved studies gave support to the notion that Vietnamese patients with depression experience and present their distress mainly through somatic symptoms. The PVPS includes a somatic scale, which accounted for a large percentage of the increase in number of detected cases of depression compared to the CIDI (2). Nguyen et al. (1) found that the most common reasons for seeking treatment among patients later diagnosed with depression were somatic complaints, namely insomnia and headaches (1). Also a study which examined the factorial structure of the Vietnamese Depression Scale among Vietnamese refugees in the USA found that there was significant overlap between the affective and somatic experiences (7). Newly arrived refugees in the USA, who were screened as depressed with the VDS had increased frequency of somatic symptoms including headaches, backaches and limb aches (11). A study assessing depression with the EPDS among postnatal women in Ho Chi Minh City (17) found that specific questions about common symptoms of depression (difficulty going to sleep, waking in the night, worrying and severe fatigue) appeared to be more meaningful to participants and were more prevalent than most non-specific somatic symptoms (e.g. difficulty swallowing, heart palpitations, breathing difficulties or heavy heart). Women with elevated EPDS scores were more likely to report gastrointestinal disturbances (17).

A Vietnamese term usually translated to neurasthenia seems to be a label that is used by Vietnamese patients and health workers to describe depression symptoms, and may be a more common reason for care-seeking than depression. This assumption is supported by a qualitative study among mothers and health workers in semi-rural Vietnam (9) and by a case study of a Vietnamese immigrant in the USA (8). In the development and use of the VDS, culture-specific symptoms were found to be frequent (12). In particular, feelings of anger, shame, and being dishonored and in despair, as well as the feeling of going crazy were important items for distinguishing those with depression, but do not fit within its Western definition (13).

A study among Vietnamese and French individuals compared the rates of depression in a community sample as obtained through International Classification of Diseases, 10^th^ revision (ICD-10) and self-rated depression in response to anchoring vignettes as none, mild, moderate, severe and extreme. The study found that being female increases the probability of self-reporting moderate, severe or extreme depression by 1.8%. This increased rate of self-rated depression among females can be explained by reporting bias – females either “over-report” or males “under-report” depression. A study among relatively socially advantaged pregnant women in Hanoi found that lack of salaried work, or a secure source of income and living in crowded conditions were associated with higher scores in the EPDS (16). Also, higher scores in the coercion subscale of the Intimate Bonds Measure (IBM) were associated with higher EPDS scores, in particular women who felt criticized over small things and who felt controlled by their partners had significantly lower mood (18). In a study among postnatal women in Ho Chi Minh City (17) women who had assistance to rest and someone to prepare special foods were less likely to be distressed. However, the avoidance of traditionally prescribed foods was associated with higher rates of distress, perhaps because it may reflect a response to critical scrutiny or active enforcement from others, in particular a mother-in-law. This study also found that having no permanent job to return to, and being unable to confide in their husbands were associated with clinically significant depressive symptomatology (17). A study among adolescents and young adults in the Chi Linh district of Hai Duong province, aged 10–24 years, found that higher depression scores in acordance with the CES-D were significantly associated with being female, unmarried, of age group 15–19 years, living in an urban area and having a higher education level (20). Finally, in a study among pregnant women in Ha Nam province, in addition to from pregnancy specific factors, experience of childhood abuse, non-economic life adversity, intimate partner violence and economic difficulties were determinants of persistent antenatal common mental disorders. The most common sources of non-economic life adversity were hostile behaviors from the in-law family, husbands having extra-marital affairs or abusing alcohol, and worrying about the health, well-being and development of ones children.

### Barriers to care-seeking and perceived causation

A lack of recognition of depression among Vietnamese was demonstrated by a study comparing depression concepts between Australian nursing students in Australia, and Vietnamese nursing students from Ho Chi Minh City (4). They were asked to respond to a vignette describing a young woman with depression symptoms, either in a family context, or merely depression symptoms listed without a context. The Australians were found to perceive the depression symptoms as much more severe than the Vietnamese, thus demonstrating differences in the recognition of symptoms as a mental health problem. The Vietnamese tended to interpret the overt expression of depression symptoms (negative emotions) as a sign of immaturity or weakness of character, and in general were less accepting of the expression of emotions (4). The tendency to not openly speak of depressive symptoms in Vietnamese society was further supported by findings from a qualitative study in a semi-rural area in the north of Vietnam (9).

A study among Vietnamese immigrants in Canada aimed to identify the barriers to care-seeking by interviewing people who were experiencing distress but not currently using clinical services (5). All the narratives of distress found here made reference to notions of ‘vital energy’ and ‘hot/cold’ and ‘wind principles’ derived from traditional Vietnamese medicine. The distress described which was most like depression was *uâ’t u’ć*, which was caused by an unacceptable social situation that one could not denounce because it was seen as socially inacceptable to express negative emotion related to social hierarchies. The symptoms were mainly handled through traditional herbal remedies, and through acceptance of the social situation through for instance spiritual means such as Buddhist meditation (5). A case study of a Vietnamese immigrant in the USA showed that depression was perceived to be caused by difficult social situations and somatic factors, rather than psychological factors (8) - a finding supported by Niemi et al. (9). A study which tested a culturally adapted form of CBT among Vietnamese refugees with PTSD and panic attacks, found it to be efficacious and acceptable. The therapy consisted of 11 individual sessions, where cultural adaptation included culturally appropriate visualization, and a form of mindfulness meditation (6).

## Discussion

We have conducted a thorough search of the existing literature concerning relevant factors for depression screening and treatment in Vietnam. We believe that we have captured a considerable part of the relevant aspects in the published scientific literature. We are, however aware of several limitations of this narrative review. For example, the versatile form of studies included does not allow quantification or pooling of the results. However, we believe that useful information was obtained and will be beneficial in guiding depression management in Vietnam.

### Implications for depression screening

Our findings implicate the importance of considering somatic symptoms when screening for depression in Vietnam. Common somatic complaints include insomnia, headache, dizziness, epigastric complaints and general aches and pains. Also, culturally adapted and dimensional screening instruments may be more sensitive in the Vietnamese setting than international, categorical diagnostic instruments. Valid cut-off scores may be lower than in most other settings [11], including neighbouring China and Thailand [12-14]. However, the Vietnamese Depression Scale, though it has been validated among refugees in the USA, has to our knowledge not yet been validated in Vietnam. Given that it appears to be a good and brief screening instrument among the refugee population, The VDS may prove useful even in the Vietnamese health care system.

The illness label ‘neurasthenia’ may be a common idiom by which depression is expressed in a Vietnamese clinical setting. The medical term *neurasthenia* is translated as Vietnamese *suy nh*ượ*c th*ầ*n kinh*, Chinese *shenjing shuairuo* or Japanese *shinkei-suijaku*, all of which also translate to the common term *nervous breakdown*. Though neurasthenia was omitted from the DSM in 1980, it is listed in an appendix as the culture-bound syndrome *shenjing shuairuo* and appears in the ICD-10. The condition is thought to persist in Asia as a culturally acceptable diagnosis, due to being less stigmatising than depression, as subjects are by definition not deranged in mind or dangerous to others [15,16]. Arthur Kleinman described Chinese neurasthenia as a culturally sanctioned idiom of distress related to depression [17], and this may be the case even in Vietnam.

### Implications for treatment planning

Many psychotherapies developed within Euro-American culture, such as CBT demand the ability and willingness to converse about private experiences and to be out-spoken about feelings and relationships [18]. Conversely, a notion of the social context of depression and the inappropriateness of indulging in one’s own emotions among Vietnamese was supported by our literature review. Traditional Confucian culture is sociocentric, where relationships with others are included in the definition of the person [19], and the self is primarily expressed through commitment to family or another social group [18]. Thus, helping a person from this cultural background to see herself as an individual caught in oppositions to the will of others could cause her to become alienated from her own family [18]. Such fundamental values that patients hold must be taken into account in order for any psychotherapy to be effective. Central features that we found of importance for treatment planning included the perceived social causation of depression, as well as the importance of accepting social hierarchies and life conditions as they are. Studies in China, where Confucian, socieocentric values are similarly predominant, have shown that individual therapies where families are not allowed to participate may be thought of as strange [20]. This may also be the case for some Vietnamese patients.

Noting that our review brought to light the importance of traditional medical frameworks for the Vietnamese understanding of, and treatment seeking for depression, TVM practitioners may prove a good resource for the management of depression. The World Health Assembly of 2009, and the Beijing Declaration on Traditional Medicine [21] indeed urge member states to consider including traditional medicine into their health systems based on local priorities and capacities as well as on evidence of safety, efficacy and quality [22]. Vietnam is among the only four countries of the world where an integrative model of health care, including traditional medicine has been enacted. This means that TVM is included in the national drug policy; providers and products are registered and regulated; traditional therapies are available at hospitals and clinics; treatment with TVM is reimbursed under health insurance; relevant research is undertaken; and education in traditional medicine is available [23]. However, in the particular area of mental health care provision, this integrative process has not been implemented. Nevertheless, there are certain barriers to the use of traditional medicine - not least the lack of agreement on what constitutes scientific evidence since the epistemologies of traditional medicine differ so vastly from that of biomedicine [24].

Additionally, the mental health care system may be strengthened by including meditation practices in to treatment plans, as such an approach may prove coherent with local understandings of depression. Indeed, mindfulness meditation has in recent years gained a growing evidence-base for depression treatment [25]. The influence of Buddhism on Vietnamese culture is vast, and it is the *Pure Land* sect of Mahayana Buddhism that is practiced by the majority. This form of Buddhism does not focus on meditation practice, but rather on ritual practices such as prayer and almsgiving [26]. However, in the recent years, meditation halls where lay people go to practice mindfulness meditation have begun to open in conjunction with much visited pagodas [27].

Finally, our review revealed a number of social aspects that are implicated in depression causation. These included problems with the in-law family, and the husband, in terms of coercion and hostile behavior. Also, job insecurity and economic difficulties were found to be determinants of depression. A UNFPA-WHO international meeting on maternal mental health in Hanoi 2007 came to the conclusion that an approach to mental health management should involve multiple sectors including those dealing with development, poverty reduction, human rights, social protection, violence prevention, education, gender, and security [28,29].

## Conclusions

Our findings indicate the importance of considering somatic symptoms when screening for depression in Vietnam as well as the use of culturally adapted and dimensional screening instruments. Our study confirms that depression reflects chronic social adversity, and thus an approach to mental health management that focuses solely on individual pathology will fail to address its prevalent social causes [29]. Further studies should elucidate whether neurasthenia is a commonly used illness label among Vietnamese patients, that coincides with depression. The tendency among Vietnamese to seek TVM and meditation practice when experiencing emotional distress was supported by our findings. In other resource scarce settings, traditional medical treatment has been found to be frequently sought by those with mental illness [30], which may be true even for the Vietnam setting, in which case such provision should be explored.

## Competing interest

The authors declare that they have no competing interest.

## Authors’ contributions

MN conceived the study, carried out literature searches and analyses, and drafted the first version of the manuscript. MM and KBG were involved in the design of the study, as well as the collection of material for analysis. PA was involved in study design and supervised the data analysis. TF conceived the study together with MN and was involved in the interpretation of results. All authors read and approved the final manuscript.
